# Optically driven liquid crystal droplet rotator

**DOI:** 10.1038/s41598-022-21146-y

**Published:** 2022-10-05

**Authors:** Keita Saito, Yasuyuki Kimura

**Affiliations:** Department of Physics, School of Science, Kyushu University, Fukuoka, 819-0395 Japan

**Keywords:** Condensed-matter physics, Materials for optics, Soft materials

## Abstract

In this study, the rotation of liquid crystal droplets induced by elliptically polarized laser light was investigated using optical tweezers. The rotation mechanism was analyzed based on the arrangement of liquid crystal molecules within the droplets. The change in the rotation behavior of nematic liquid crystal (NLC) droplets was evaluated by varying the droplet size. The experimental results were analyzed based on the waveplate effect and light-scattering process. The rotation behavior of cholesteric liquid crystal droplets was examined by varying the droplet size and helical pitch, which was controlled by the chiral dopant concentration. The results are discussed in terms of the selective reflection of the incident beam by the helical structure. The dependence of the rotation frequency on the ellipticity of the incident beam was also studied. The main contribution to the rotation gradually changes from light transmission to reflection with increasing chirality of the droplet. An NLC rotator system was constructed using holographic optical tweezers. Such an optically controllable rotator is a typical micro-optomechanical device. Complex flow fields, including multiple vortex and localized shear fields, were realized at the micron scale.

## Introduction

Manipulation of materials at the microscale is crucial for evaluating the microscopic properties of soft materials and biomaterials^1,2^. Optical tweezers are vital tools to precisely control micro-objects, such as colloids, microorganisms, and cells^3^. The linear and angular momenta of light drive their translational and rotational motions, respectively. For instance, colloids can be arranged in complex patterns and dynamically driven in complex ways^4^. The orientation of birefringent objects can also be controlled using polarized light^5^. In particular, the irradiation of a birefringent object with circularly polarized light induces continuous rotation (spinning motion)^5^.

Liquid crystal (LC) droplets are typical birefringent materials that can be rotated by circularly polarized light^6–8^. Their inner structure depends on the boundary conditions of the molecules on the droplet surface^9,10^. For tangential anchoring at the surface of a nematic LC (NLC) droplet, the LC molecules are aligned parallel to the droplet surface, and two point defects exist at the poles of the droplet; this is called the bipolar structure^10^. For homeotropic anchoring, the NLC molecules are arranged radically, and a single-point defect exists at its center; this is known as the radial structure^10^. In addition to the bipolar and radial structures, several other structures exist, depending on the strength and type of anchoring^10^. A cholesteric LC (ChLC) droplet can be formed by stirring the mixture of an NLC and a chiral dopant^10,11^. ChLC droplets have a helical molecular arrangement. The ratio of the droplet diameter *d* to helical pitch *p* is a critical parameter that determines the inner structure of the droplet^11^.

Several mechanisms for the rotation of LC droplets have been investigated, and their main contributions depend on their inner structure^12–18^. For example, in a bipolar structure, the waveplate effect and light-scattering process are dominant^13–15^, and the rotation frequency reaches up to 10^3^ Hz^6^. However, the droplet does not rotate under weak light in a radial structure and does not induce a change in the inner structure^7^. In chiral solid particles composed of optically cured ChLCs, the Bragg reflection induced by the helical arrangement of LC molecules is the main contributor to rotation by Gaussian^16,17^ and non-Gaussian trap^18^. Because Bragg reflection only occurs when the direction of circularly polarized light is the same as the chirality of the particle, the chiral particle rotates only in the same direction as the chirality^16^. Under specific conditions (strong light irradiation that reorganizes the molecular alignment of a ChLC droplet with $d/p$ = 0.5 or 1), linearly polarized light induces droplet rotation^19^.

LC droplet is useful for opto-microfluidic device because rotation velocity can be controlled without contact and size of the droplet can be easily controlled. Increasing energy transfer efficiency of LC droplet is important to apply LC droplet to opto-microfluidic devices. It is necessary to establish the relationship between the optical interactions of the LC droplets and their inner structures to clarify the rotation mechanism in detail. This clarification will facilitate the design of devices with high energy transfer efficiency. In addition, the rotating microparticles induce a local flow field around them, generating complex flow fields at the microscale. This technique will motivate further investigations of the complex phenomena caused by local vortex flow and shear stress in soft matter^20^.

In this study, we investigated the optical torque transfer to NLC and ChLC droplets with changes in the inner structure to understand which inner structure rotates LC droplet more efficiently. The effects of droplet size and chirality on the applied torque were measured, and the experimental results were analyzed based on the inner structure of the droplets^21,22^. From an application viewpoint, a controllable rotating NLC droplet system was constructed using a holographic optical tweezer (HOT)^23^.

## Results and discussion

### Rotation of NLC droplet

#### Dependence of rotation on laser power and ellipticity angle of polarized laser beam

**Figure 1:**
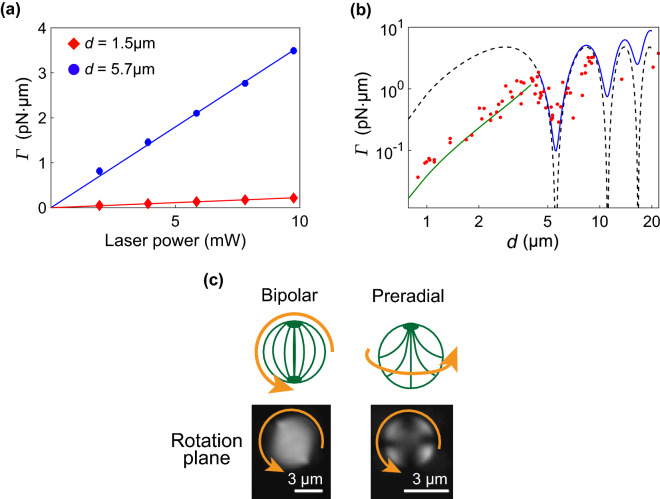
Optically induced continuous rotation of NLC (E7) droplet. (**a**) Variation in optical torque *Γ* applied to droplet with laser power. The solid lines are the best-fitted ones to data. (**b**) Variation in optical torque *Γ* with diameter *d* of droplets. The dashed line is the best-fitted curve of Eq. (3). The green and blue lines are the best-fitted curves of Eq. (4) for *d* < 4.5 µm and *d* > 4.5 µm, respectively. (**c**) Polarizing microscopic images of NLC droplet under crossed-nicols polarizers and the schematics of molecular alignment. The molecular alignment within a smaller droplet is preradial, and that within a larger droplet is bipolar. The arrows indicate the rotation directions.

When a droplet exhibits stationary rotation with frequency *ν*, the optical torque *Γ* received from the laser beam is balanced by the viscous torque from the solution as (see Supplementary Information)^24^

1 $${\it \Gamma} =\pi \eta \nu {d}^{3},$$

where *η* is the viscosity of water, and* d* is the diameter of the droplet. The relationship between *Γ* estimated using Equation (1) and the laser power is depicted in Fig. 1a. *Γ* linearly increased with increasing laser power from 1.9 to 9.4 mW.

Optical torque *Γ* also depends on the ellipticity angle, *φ*, of the laser beam. The linearly polarized beam corresponds to *φ* = 0, and the circularly polarized beam corresponds to *φ* = π*/*4. When the polarization of the trapping beam approached circular polarization above a certain critical value |*φ*_c_|, the NLC droplet started to rotate, and *ν* increased with increasing |*φ*|^5,7^. This behavior is explained by the waveplate effect^5,7^. The optical torque *Γ* at *φ* is expressed as

2 $${\it \Gamma} =\frac{P}{\omega }\left[\left(1-\cos\Delta \right)\sin\;2\varphi -\sin\;\Delta\; \cos \,2\varphi \sin\;2\theta \right],$$

where *P* is the laser power, *ω* is the frequency of the trapping beam, ∆ is the retardance expressed by ∆ = 2*π*∆*nd/λ* (∆*n* is the birefringence, and λ is the wavelength of the trapping beam), and *θ* is the angle between the major axis of the polarization ellipse and the optical axis of the object. The first term on the right-hand side of Eq. (2) represents the contribution of the spin angular momentum, which rotates the object in the same direction as the rotation for the elliptical polarization. The second term represents the alignment effect along the major axis of the elliptical polarization, except if the incident beam is circularly polarized.

#### Size dependence of optical torque

The dependence of *Γ* on diameter *d* of the NLC droplets is complex, as shown in Fig. 1b. We analyze the size dependence by dividing it into two regions. For *d* < 4 µm, *Γ* increased monotonously with *d*. For *d* > 4 µm, *Γ* exhibited oscillatory behavior. There are four proposed origins of optical torque^14^: the waveplate effect, light-scattering process, photon absorption, and light-induced Fréederickzs transition. Only the optical torque generated by waveplate effects showed oscillatory dependence on droplet size, and its magnitude depended on the retardation, Δ = 2*π*∆*nd/λ*, of the droplet^7,14^. The oscillatory behaviors in different NLCs, 5CB and E7 scaled with ∆*nd* (see Supplementary Information). This trend also supports the concept that the waveplate effect significantly contributes to the optical torque for large droplets.

The waveplate effect in a spherical birefringent particle was evaluated to quantitatively determine the variation in *Γ* with *d*. For steady-state rotation, the average optical torque *Γ*_ave_ generated by the waveplate effect is obtained by calculating the azimuthal average of Eq. (2) within $0\le \theta \le 2\pi$^5^ as ${\it{\Gamma }}_{{\rm ave}}=\frac{P}{\omega }\left(1-\mathrm{cos\;\Delta }\right)\mathrm{sin}\;2\varphi$. When ∆*n* is independent of *d*, *Γ*_ave_ depends only on *d*. Therefore, we decomposed the irradiated area into hollow cylinders and estimated the total torque *Γ*_WP_ for circularly polarized light (*φ* = π/4) by summing their respective contributions (see Supplementary Information) as

3 $${\it{\Gamma }}_{\mathrm{WP}}=\frac{\pi{\mathcalligra{p}}{d}^{2}}{2\omega }\left[\frac{1}{4}\left(1-\mathrm{cos}\;2{\theta }_{1}\right)+\frac{1}{{\Delta }^{2}}\left({t}_{1}\mathrm{sin}\;{t}_{1}+\mathrm{cos}\;{t}_{1}-\mathrm{\Delta sin\;\Delta }-\mathrm{cos\;\Delta }\right)\right],$$

where ${\mathcalligra{p}}$ is the power density of the laser, *θ*_1_ is the azimuth angle indicating the beam diameter (beam size = *d*sin*θ*_1_) and *t*_1_ = Δcos*θ*_1_. The beam diameter is approximately 1*.*22*λ/*NA, where NA is the numerical aperture. The best-fitted line of Eq. (3) with ∆*n* = 0.192^25^ and the fitting parameter ${\mathcalligra{p}}$ for the experimental data is shown as a dashed line in Fig. 1b. The line captures the oscillatory behavior of large droplets, but a clear discrepancy exists with the experimental data for small droplets. This difference partly occurred because only the waveplate effect was considered. Previous studies have suggested that the waveplate effects and light-scattering process mainly contribute to the rotation of NLC droplets^14^. However, the relative contribution of two effects was not quantitatively mentioned. Because the optical torque significantly depends on the inner structure of the droplet, molecular alignment was observed within the LC droplets. The inner structure changed at approximately *d* = 4 µm. In larger droplets (*d* > 4.5 µm), the inner structure was bipolar (Fig. 1c, left), and *Γ* oscillated in this region. However, for *d* < 4.5 µm, the inner structure changed to a preradial structure (Fig. 1c, right), and *Γ* was proportional to *d*^3^. Such changes in the inner structure have also been reported in a previous study^21^. The critical size between the preradial and bipolar structures coincided with the size at which the dependence of *Γ* on *d* changed in our experiment. The power dependence in Fig. 1a is linear for both bipolar and preradial droplet. For a radial droplet, the nonlinear power dependence has been reported^7^. The linear dependence indicates that local optical deformation is not induced in a preradial droplet at lower power we used.

The optical torque *Γ*_all_ reflecting the torques generated by the waveplate effect *Γ*_WP_ and light-scattering process *Γ*_LS_ is expressed as

4 $${\it{\Gamma }}_{\rm all}=a{\it{\Gamma }}_{\mathrm{WP}}+\left(1-a\right){{\it\Gamma }}_{\mathrm{LS}},$$

where *a* is the ratio of *Γ*_WP_ to *Γ*_all_ (0 ≤ *a* ≤ 1). *Γ*_LS_ is expressed as^12,14^ ${{\it \Gamma }}_{\rm{LS}}=\frac{\alpha \mathcalligra{p}n}{c}V$, where *V* is the droplet volume, *n* is the average refractive index, *c* is the speed of light in vacuum and *α* is the angle between the Poynting vector of light and the wave vector, derived from ∆*n* and *n* (see Supplementary Information)^12,14^. The experimental data is separately fit with Eq. (4) at *d* < 4.5 µm and *d* > 4.5 µm, where ${\mathcalligra{p}}$ and *a* are the fitting parameters. The green and blue lines in Fig. 1b represent the best-fitted curves of Eq. (4) to data for *d* < 4.5 µm and *d* > 4.5 µm, respectively. For *d* > 4.5 µm, the optimal value of *a* was 0.997, and the waveplate effect was dominant. This finding is consistent with the fact that the inner bipolar structure is anisotropic, and the waveplate effect is the dominant contributor. For *d* < 4.5 µm, *a* was 0.24, and the light-scattering process was dominant. In the rotation plane, the preradial structure was more isotropic than the bipolar structure. Therefore, the contribution of the waveplate effect was less significant than that of the light-scattering process. The energy efficiency, defined as the ratio of the power estimated by the fitting to that measured by the power meter at the focal plane, was six times higher for bipolar droplets than for preradial droplets (9% and 1.5% for bipolar and preradial droplets, respectively). It was confirmed that the NLC droplet with a bipolar structure converted optical energy into mechanical energy more efficiently than that with a preradial structure. Considering waveplate effect and scattering one based on the birefringent model, the contribution of two effects and the energy efficiency were quantitatively discussed.

### Rotation of ChLC droplet

#### Dependence of rotation on ellipticity angle of incident beam

Based on the above discussion, the inner structure of the LC droplet is crucial to its rotational mechanism. We introduced helical modulation in the arrangement of LC molecules in NLC droplets by adding a chiral dopant. The amount of chiral dopant determines the optical chirality of ChLC droplets.

**Figure 2:**
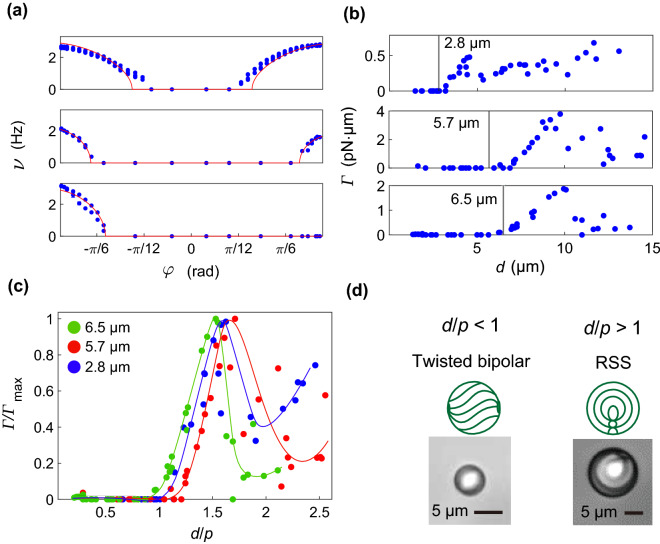
Optically induced continuous rotation of ChLC (E7+R-811) droplet. (**a**) Variation in rotation frequency *ν* with ellipticity angle of polarized light *φ* with 10 mW laser power for NLC droplets and 25 mW for ChLC droplets. The R-811 concentrations are 0 wt% (top), 0.3 wt% (middle), and 1.0 wt% (bottom). The red lines are theoretical curves fitted using Eq. (5). (**b**) Variation in applied torque *Γ* with diameter *d* for three ChLC droplets with different amount of R-811 (upper: 4.6 wt%, middle: 0.7 wt%, and lower: 0.1 wt%). The vertical line indicates pitch *p* of ChLC. 17.5 mW laser power was used. (**c**) Variation in scaled optical torque *Γ*/*Γ*_max_ with scaled diameter *d/p*. The solid lines are eye guides. (**d**) Bright field microscopic images and schematically shown molecular alignment of ChLC droplets with twisted bipolar structure and RSS. For *d/p* < 1, the inner structure is twisted bipolar; for *d/p* > 1, the inner structure is RSS.

The change in rotation of the NLC and ChLC droplet was investigated by varying the ellipticity angle *φ* of the incident beam. Two droplets with different concentrations of the chiral dopant R-811 (0.3 and 1.0 wt%) were prepared. Figure 2a shows the variation in rotation frequency *ν* with *φ*. For an NLC droplet, the dependence is almost symmetric with respect to *φ* = 0, and the direction of rotation was determined by that of the circularly polarized beam (Fig. 2a, top). In contrast, the dependence on *φ* was asymmetric for the ChLC droplets, and *ν* for the beam with chirality opposite to ChLC decreased as the amount of chiral dopant increased. Chiral solid particles have been reported to selectively reflect circularly polarized light with chirality similar to that of a particle^16,17^. In the lower-chirality region, as in our ChLC droplets, transmission and reflection of the circularly polarized beam co-occurred. When the torque induced by reflection and transmission, *ν* is written as^17^

5 $$\begin{aligned} \nu & = \frac{P\lambda }{{4\pi^{3} c\eta d^{3} }}{\text{Re}}\left[ {\left\{ {{\text{sin}}^{2} 2\varphi \left[ {\left( {1 + \frac{R}{2}} \right) - \left( {1 - \frac{R}{2}} \right)\cos {\Delta }} \right]^{2} + R\left( {1 + \cos {\Delta }} \right)\sin 2\varphi } \right.} \right. \\ & \quad \left. {\left. { \times \,\left[ {\left( {1 + \frac{R}{2}} \right) - \left( {1 - \frac{R}{2}} \right)\cos {\Delta }} \right] + \frac{{R^{2} }}{4}\left( {1 + \cos {\Delta }} \right)^{2} - \left( {1 - R} \right){\text{cos}}^{2} 2\varphi {\text{sin}}^{2} {\Delta }} \right\}^{\frac{1}{2}} } \right], \\ \end{aligned}$$

where *R* is the reflectance of the circularly polarized light in the same rotational direction as the chirality of the ChLC droplet. The results obtained using Eq. (5) are consistent with the experimental data, as indicated by the solid lines in Fig. 2a. The best-fitted value of *R* increased with increasing amount of R-811 (*R* = 0%, 4%, and 8% for 0 wt% [NLC], 0.3 wt%, and 1.0 wt% of R-811, respectively). The chiral dopant induces a helical structure, and the reflectance of circularly polarized light from the helical structure depends on the degree of chirality.

#### Size dependence of optical torque

The variation in *Γ* with diameter *d* of the ChLC droplets subjected to right-handed circularly polarized light is shown in Fig. 2b. Three ChLCs droplets were prepared using R-811 concentrations of 0.1, 0.7, and 4.6 wt%, respectively. The inner structure of ChLC droplets depends on the ratio of *d* to the pitch of the helical structure, *p*^26^. In Fig. 2c, *Γ* is normalized by its maximum value *Γ*_max_, and *d* is normalized by *p*. The overall dependence trend was consistent for the three ChLCs. For *d*/*p* < 1, the ChLC droplets did not rotate within the laser power range used. In this case, the inner structure of the droplet was twisted bipolar (Fig. 2d, left) and acted as a waveguide^27^. Therefore, the droplet did not change the polarization of the incident light, and no angular momentum transfer from the light to the droplet occurred. For *d*/*p* > 1, the inner structure changed to a radial spherical structure (RSS) (Fig. 2d, right), and the droplet rotated. In the RSS, the function of the waveguide vanished, and angular momentum transfer occurred. All plots had a peak at approximately the same position (*d*/*p* ~ 1.5). At this peak position, *d* approaches the Bragg wavelength *np* of the ChLC droplet, where *n* is the refractive index of approximately 1.503 for E7^25^.

### Multiple rotating LC droplet system

We constructed a microscale device to control the local flow field using rotating NLC droplets. In our system, the spatial arrangement of NLC droplets can be controlled by the HOT. The rotation speed and direction of droplet rotation can be controlled by the laser power and the circular polarization direction, respectively.

**Figure 3:**
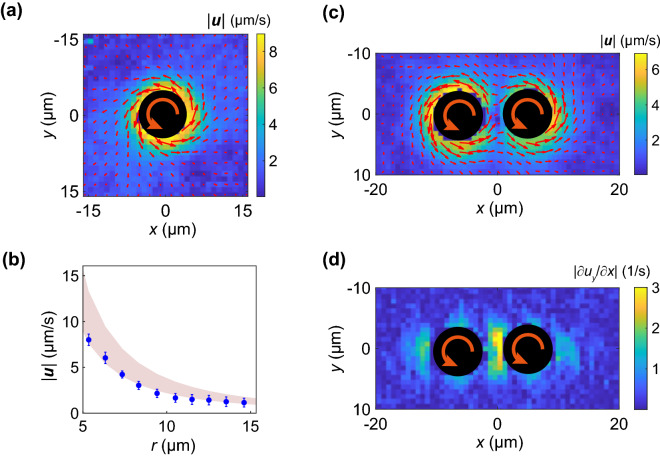
Flow field induced by NLC droplet rotators. (**a**) Velocity field generated by rotation of single NLC(E7) droplet with radius of 3.6 ± 0.3 µm. The arrows represent local velocity vector. The laser power used was 29 mW. (**b**) Variation in azimuthal component of induced velocity field ${\varvec{u}}$ in (**a**) with distance *r* from center of rotating NLC droplet. The shaded area shows the range of the velocity estimated using Eq. (6). (**c**) Induced velocity field ***u*** for two rotating NLC droplets with 36 mW laser power. The arrows represent local velocity vectors. (**d**) Shear rate of flow velocity in y-direction $\frac{\partial {u}_{y}}{\partial x}$ evaluated from (**c**).

The flow field induced by droplet rotation was measured using microparticle image velocimetry^28^. Silica particles with diameters of 1 µm were dispersed in the solution as probes. Because most particles settled at the bottom of the cell, a rotating NLC droplet was placed close to the bottom. Figure 3a shows the flow field around a single rotating NLC (E7) droplet. The flow velocity induced by a rotating solid spherical particle with diameter *d* is expressed as^29^

6 $${\varvec{u}}=\frac{{d}^{3}}{8{r}^{3}}\left(2\pi{\varvec{\nu}}\right)\times {\varvec{r}},$$

where ***r*** is the distance vector from the center of the particle, ***ν*** is the angular frequency vector of the particle, and ***u*** is the azimuthal flow velocity at position ***r***. The azimuthal component of the average flow velocity obtained experimentally and that estimated using Eq. (6) are shown as the shaded area of Fig. 3b. The measured values are consistent with the theoretical values, confirming that the NLC droplet behaves as a solid particle.Multiple optical trapping sites can be designed using HOT. Two droplets of approximately the same size were trapped using optical tweezers, and the flow field around them was measured (Fig. 3c). Because the direction of the rotation was the same, a circulating flow appeared around the two particles, and a shear field was induced in the gap between the droplets (Fig. 3d). The intensity and spatial arrangement of the shear field could be controlled by the rotation of the NLC droplets with the HOT. This technique enables the realization of instantaneous complex microflow fields in microfluidic devices, leading to further possibilities for research on the mesoscopic properties of soft matter.

## Conclusion

By changing inner structure of droplets and considering each effect, this study reveals relative contribution of each effect to the rotation and the energy transfer efficiency, which are not mentioned in previous works. In NLC droplets, the waveplate effects and light-scattering process mainly contribute to rotation. When the diameter is larger than the critical size (approximately 4.5 µm in this study), the inner structure is bipolar and optically anisotropic. In this case, the main contribution is the waveplate effect, and bipolar droplets have the highest energy transfer efficiency in prepared droplets. Therefore, bipolar droplets are appropriate for opto-mechanical device. As the diameter of the NLC droplet decreases, the optical anisotropy of the inner structure decreases, and the inner structure becomes preradial, which is isotropic in the rotational plane. In this case, the waveplate effect weakens, and the scattering process dominates. In ChLC droplets, transmissions and Bragg reflections owing to the helical structure appear. Therefore, the angular momentum of the light is transferred to the ChLC droplet via the waveplate effect and Bragg reflection. The torque induced by the waveplate effect and Bragg reflection explains the rotational behavior influenced by the ellipticity angle, *φ*. The diameter-to-pitch ratio, *d/p*, is critical for its rotation behavior. For *d/p* < 1, the inner structure is twisted bipolar and acts as a waveguide. Therefore, the transfer from light to the ChLC droplet was minimal, and the ChLC droplet did not rotate. As an application of the rotating droplets, a controllable microflow field was constructed using the HOT. The flow field induced by the rotating single NLC droplet is consistent with the theoretical prediction. In the system with two NLC droplets, a shear field was induced in the gap between the droplets. A more complex flow field can be designed using HOT. This technique opens a new avenue for the micromanipulation of soft matter and analysis of their mesoscopic properties.

## Methods

### Optical system

In our optical system, the wavefront of the laser beam (YLM-10-CP, IPG Photonics, wavelength of 1064 nm) was modified using a spatial light modulator (SLM, X10468-03, Hamamatsu) to control the spatial intensity pattern at the focal plane. The reflected beam from the SLM was transmitted through a half-wave plate and a quarter-wave plate to control the ellipticity angle, *φ*, and orientation angle, *θ*, of the polarization ellipse of the trapping beam. Finally, the polarized light was focused using a 100 × objective lens (Plan Fluor, Nikon, NA1.4). At the focal point, LC droplets were trapped above 20 µm from the bottom of the cell to prevent the wall effect (see Supplemental Information) and were forced to rotate with the trap. The laser power used at the focal plane was 7.5 mW, measured using a power meter (PM16-405, Thorlabs) unless otherwise noted.

### Materials

We used 5CB (TCI) and E7 (a mixture of 51 wt% 5BC (TCI), 25 wt% 7CB (Sigma-Aldrich), 16 wt% 8OCB (TCI), and 8 wt% 5CT (TCI)) as NLCs. E7 and 15 wt% RM257 (Sigma Aldrich), a photo-polymerizable monomer^30^, were mixed with toluene to prepare solid birefringent particles. After the toluene evaporated, the mixture (E7 and RM257) and water were stirred to prepare the droplets. The sample dispersion was irradiated with ultraviolet light for 30 min to solidify the droplets via photopolymerization. E7 and right-handed chiral dopants R-811 (Merck) were mixed in isopropanol as a solvent to prepare ChLC. After stirring in a magnetic stirrer for 3 h, isopropanol was evaporated to prepare the ChLC. A mixture of ultrapure water (18.2 MΩ cm) and the prepared ChLC was stirred vigorously to form ChLC droplets of 1–20 μm diameter. The droplets dispersed in water were sealed in a glass cell approximately 85 μm thick.

### Rotation frequency analysis

Images of the trapped LC droplets were captured using a complementary metal-oxide-semiconductor (CMOS) camera (Orca-Flash 4.0, Hamamatsu, 2048 × 2048 pix^2^) attached to an inverted optical microscope (Eclipse Ti, Nikon). The exposure time of the CMOS camera varied depending on the droplet size, that is, 2 ms for tiny droplets (*d* < 3 µm) and 20 ms for large droplets (*d* > 3 µm). Because the polarizer and analyzer were removable, the image could be switched between bright-field and polarization. The rotational motion could be monitored from the temporal change in the image intensity, owing to the birefringence of LC droplets^13^. The time evolution data of the sum of the image intensity along a horizontal line across the center of the droplet were Fourier-transformed to determine the droplet rotation frequency *ν*. The lowest frequency peak corresponded to either the 2*ν* or 4*ν* modulation of the rotation frequency, depending on the inner structure. We watched the videos to confirm whether the low-frequency peak was either 2*ν* or 4*ν* and finally determined *ν*.

### Microparticle image velocimetry

Silica particles (sicastar-greenF, Micromod) of 1 vol% were used as tracer particles to visualize the flow field. Because the silica particles settled in the water, a rotating NLC was set at the bottom of the cell. We recorded a video at 200 frames/s under bright-field conditions. The video was analyzed using PIVlab^31^ to calculate the flow field.

## Supplementary Information

Supplementary Information.

## Data Availability

The datasets generated during and/or analyzed during the current study are available from the corresponding author on reasonable request.
