# Supplementary material for: Optically driven liquid crystal droplet rotator
Source: Sci Rep. 2022 Oct 5;12:16623. doi: 10.1038/s41598-022-21146-y (PMC9534986; doi:10.1038/s41598-022-21146-y)
Supplement: Supplementary file 1 — Supplementary Information. [file 41598_2022_21146_MOESM1_ESM.pdf]

Supplementary Information for
"Optically driven liquid crystal droplet rotator"

Keita Saito[1] and Yasuyuki Kimura[2]*

[1,2]Department of Physics, School of Science, Kyushu University, 819-0395 Fukuoka, Japan

E-mail addresses: [1] saitodes3110des@gmail.com, [2] kimura@phys.kyushu-u.ac.jp

*Corresponding author

Comparison of rotation frequency of NLC droplets and their optically cured solid particles

We compared the rotation frequency, v, of NLC droplets and their optically cured solid particles. In our experiment, we used a mixture of E7 and the photopolymerizable monomer, RM257 (15wt%, Merck[1]). Supplementary Fig. 1 shows the variation in v with size d for the NLC droplets and cured solid particles. The fluidity of the LC droplet had no significant effect on the rotational frequency because the viscosity of NLC was significantly higher than that of the surrounding water (the viscosity of E7 is approximately 100 times higher than that of water[2]). Therefore, in a steady state, the optical torque, Γ, is equal to the viscous torque of a solid sphere, expressed by Equation (1)[3] in the main text.

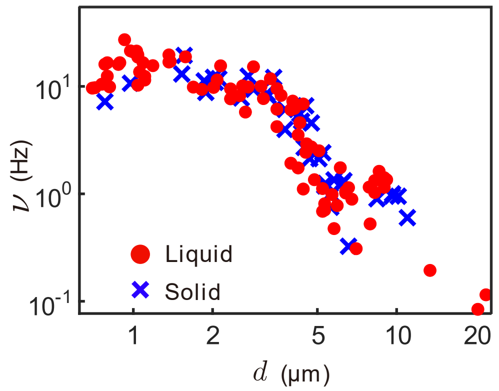

Supplementary Figure 1. Variation in rotation frequency *v* with diameter of particles *d* (E7-RM257 mixture). ●: NLC droplets, ×: polymerized particles

Comparison of size-dependent torque for different NLC droplets

Supplementary Fig. 2 shows the variation in Γ with Δnd for the E7 and 5CB droplets. The birefringence Δn of E7 and 5CB were 0.192 and 0.159, respectively[4]. The overlap between

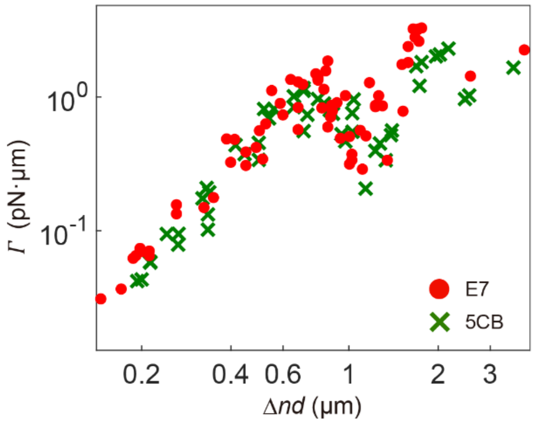

Supplementary Figure 2. Variation in Γ with Δnd. ●: E7, ×: 5CB.

the E7 and 5CB data indicates oscillatory behavior. Thus, the waveplate effect is the dominant contributor to Γ.

Wall effect on LC rotation

We determined the variation in the rotation frequency, ν, of an NLC droplet (E7) with its distance from the bottom wall to analyze the effect of the cell wall on its rotation. ν decreased as the droplet approached the bottom (Supplementary Fig. 3), and ν reached the minimum value when the droplet was in close contact with the wall of the cell ($z = z_0$, z: z-stage position). When $z - z_0 > d$ (d is the diameter of the droplet) for bottom case, the wall effect on droplet rotation was negligible. During the entire experiment, the trapping position was set at approximately 20 μm above the bottom where the position is approximately 65 μm below the top of the cell. When the droplet was pressed against the bottom, ν increased rapidly. In this case, the focal point was below the center height of the droplet, and the shape of the droplet was deformed. In

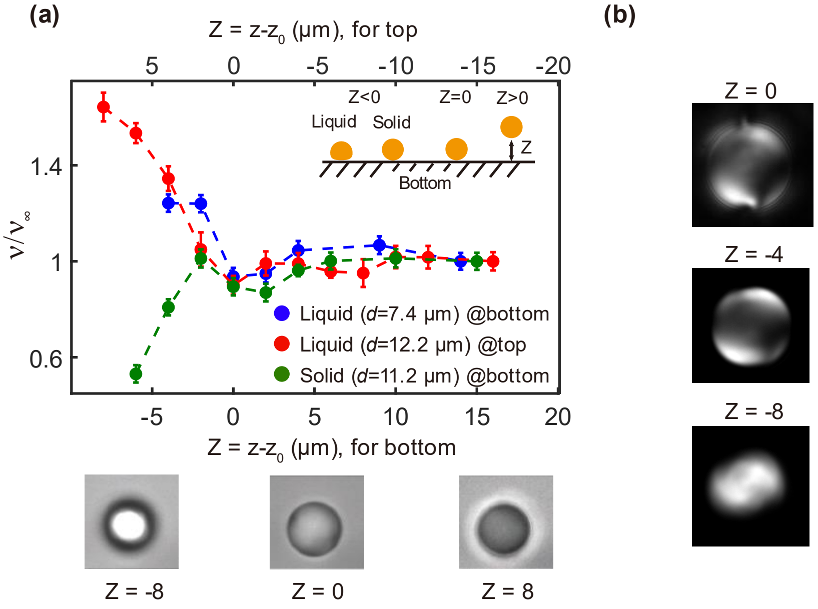

Supplementary Figure 3. Variation in rotation frequency ν of droplet with distance from cell wall. (a) z is the z-stage position, and z_0 is the position where a droplet touches the bottom of the glass cell. For the bottom case, in region $Z = z\text{-}z_0 > 0$, Z is the gap between the cell and the droplet. In $Z < 0$, a droplet pushes against the bottom wall, deforming its shape. For the top case, a droplet pushes against the wall in $Z > 0$, and $|Z|$ is the gap between the cell and the droplet in $Z < 0$. Bright-field microscopic images of a droplet are shown in the bottom row. At $Z = -8$ μm, the white area corresponds to the region where the droplet touches the wall. (b) Polarizing microscope images of droplet at different z-position. The images were captured under cross-Nicol state.

upper wall case, the dependence of v on z-position is similar to the bottom wall case. Because the anisotropy of the inner structure of the droplet is critical for the transfer of angular momentum[5], we observed the inner structure of the deformed droplet under crossed-Nicol state. The change in the inner structure depending on z-position was found as shown in Supplementary Fig. 3(b). This result indicates that the deformation changes the birefringence. As an additional validation of deformation, the same measurement with polymerized droplet which does not deform was performed. In polymerized droplet case, the rotation frequency decreases when the droplet is pressed as shown in Supplementary Fig. 3(a). This result supports that the deformation is important to increase the rotation frequency. This can be applied to measure the sliding drag coefficient between the droplet and solid wall.

Application of waveplate effect to partially irradiated sphere

In our experiment, an LC droplet was partially irradiated, as shown in Supplementary Fig. 4. The irradiated area, S, was decomposed into hollow discs, and the birefringent sphere was decomposed into hollow cylinders. The optical axes of the cylinders were perpendicular to the center axis. The optical torque exerted on a hollow cylinder, $d\Gamma$, was estimated based on the waveplate effect as $pdS/\omega(1-\cos\Delta)\sin 2\varphi$, where Δ is the retardance of the hollow cylinder ($\Delta = 2\pi\Delta nl/\lambda$, l is the height of the hollow cylinder), dS is the irradiated area of the hollow cylinder, and p is the power density of the laser (pdS corresponds to P in Γ_{ave}). In circularly polarized light ($\varphi = \pi/4$), we estimated the total torque, Γ_{WP}, by summing $d\Gamma$ as

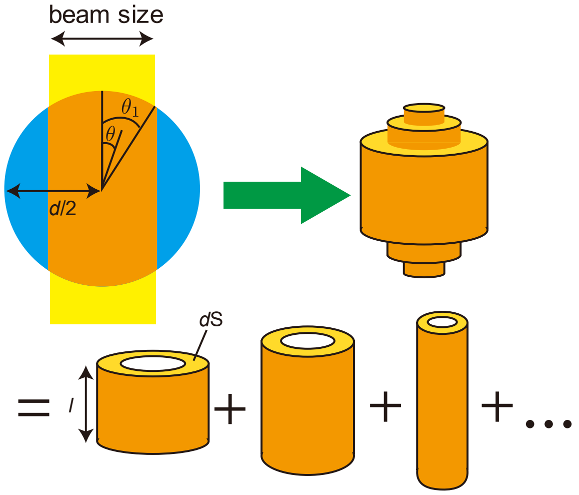

Supplementary Figure 4. Schematic of trapped droplet. The orange region in the droplet represents the irradiated region by the incident beam. The irradiated region is decomposed into hollow cylinders.

$$\Gamma = \int d\Gamma = \int_0^{\theta_1} \frac{p}{\omega}(1-\cos\Delta)2\pi\left(\frac{d}{2}\right)^2 \sin\theta\cos\theta\, d\theta$$

$$= \frac{\pi p d^2}{2\omega}\left\{\frac{1}{4}(1-\cos 2\theta_1) + \frac{1}{\Delta}(t_1\sin t_1 + \cos t_1 - \Delta\sin\Delta - \cos\Delta)\right\},$$

where θ is the azimuth angle, $d\sin\theta_1$ is the beam size, and $t_1 = \Delta\cos\theta_1$, as shown in Supplementary Fig. 4.

Estimation of angle between Poynting vector and wave vector α

We estimated the angle between the Poynting vector and wave vector to determine the magnitude of the light-scattering process. In a uniaxial crystal, α is expressed as[6]:

$$\tan\alpha = \frac{1}{2}n_{\mathrm{eff}}^2(\theta)\left(\frac{1}{n_e^2}+\frac{1}{n_o^2}\right)\sin 2\theta,$$

$$n_{\mathrm{eff}}(\theta) = \frac{n_o n_e}{\sqrt{n_o^2\sin^2\theta + n_e^2\cos^2\theta}},$$

where n_o is the ordinary refractive index, n_e is the extraordinary refractive index, and θ is the angle of incidence. In our case, θ ranged between 0 and 1.17 because we used focusing light with a numerical aperture of 1.4, and the refractive index of immersion oil is 1.518. Each α value for different incident angles ($0 < \theta < 1.17$) was calculated, and the average value of α was used to estimate the magnitude of the light-scattering process.

Acknowledgements

We thank anonymous referee for valuable comments and suggestions regarding wall effects.

Supplementary references

[1] Wang, X., Bukusoglu, E. & Abbott, N. L. A practical guide for the preparation of liquid-crystal-templated microparticles. *Chem. Mater.* **29,** 53–61 (2017).

[2] Basu, R., Kinnamon, D., Skaggs, N. & Womack, J. Faster in-plane switching and reduced rotational viscosity characteristics in a graphene-nematic suspension. *J. Appl. Phys.* **119,** 185107 (2016).

[3] Happel, J. & Brenner, H. *Low Reynolds number hydrodynamics: with special applications to particulate media* (Springer, 2012).

[4] Tkachenko, V. *et al.* Nematic liquid crystal optical dispersion in the visible-near-infrared

range. *Mol. Cryst. Liq. Cryst.* **454,** 263/[665]–271/[673] (2006).

[5] Wood, T. A., Gleeson, H. F., Dickinson, M. R. & Wright, A. J. Mechanisms of optical angular momentum transfer to nematic liquid crystalline droplets. *Appl. Phys. Lett.* **84,** 4292–4294 (2004).

[6] Yariv, A. & Yeh, P. *Optical waves in crystals: Propagation and control of laser radiation* (Wiley-Interscience, 1983).
